# Mode of Origin of Some Secondary Lesions

**Published:** 1881-08

**Authors:** Edward W. Cox Moore


### ARTICLE V.

### *On the Mode of Origin of Some Secondary Lesions.*

(A paper read before the Students' Society of the National Dental Hospital and College, March 11, 1881.)

BY EDWARD W. COX MOORE, L. D. S. R. C. S. I.

*Mr. President and Gentlemen.*—In most books on Dental Surgery a considerable amount of space is usually devoted to a description of the nervous affections which may result from the condition of the teeth, these diseases occurring in some cases where a healthy tooth is undergoing normal development, and in others where we have to deal with a tooth which has undergone decay, on one in which some new growth presses upon the nerves in connection with the tooth. This subject is one which it is difficult to handle from a purely Dental point of view, owing to the various structures which have to be brought under consideration and the variety of diseases that may affect those different structures.

The object of this paper is not to give a *resume* of the multiform diseases resulting from irritation in and about the teeth, but rather to investigate the question as to the mechanism by which some of the more complex cases of reflex irritation, with the resulting lesions, can be explained and shown to be parallel to the mode of production of other diseases in the body. It will perhaps simplify matters to commence by giving a few examples of the special form of secondary disease which it is the object of this paper to elucidate.

The first example we take is one related by Mr. Hilton of the late celebrated Dr. Addison.

"The latter was suffering from an offensive discharge from the auditory canal of one of his ears, which annoyed him very much, and below the external ear was a small gland enlarged in the upper part of the neck. He had tried various remedies for this discharge, and had gone, I believe, to some surgeons who made a specialty of diseases of the ear, but as far as I could judge ongood resulted from any of their applications. Upon examining the ear from which the offensive discharge proceeded, I found a slight ulceration upon the floor of the auditory canal. On arguing the question out between us, we came to the conclusion that the ulceration probably depended upon a diseased molar tooth in the lower jaw on the same side. We had that tooth extracted, and in a very short space of time the ulcer healed, the discharge and morbid secretion disappeared from the auditory canal, and so soon as that ulceration was cured, the enlarged gland subsided."

In this case the symptoms, though troublesome, were not dangerous: but we pass on next to a class where more important structures are affected, so ably described by Dr. Woakes in his work on "Deafness, Giddiness, and Noises in the Head," viz:

"A child is cutting its teeth, and while the gums are yet swollen it suffers acutely from earache; any one accustomed to watch carefully the symptoms of these little patients will scarcely fail to discern in the troubled face, the thrill of agony, accompanied with cries or shrieks, when its position is moved, and above all the constant raising of the hand to the side of the head; no one who has watched these symptoms will fail to connect them with the most agonizing sufferings of early life—'earache.'

"On examining under these circumstances with the speculum, in a good light, the drum of the ear, it will be found to have exchanged its pearly-like lustre for one of redness; this gradually extends to the lining membrane of the cavity of the tympanum, and unless this condition be actively treated by lancing the gums and local removal of blood, the further stage is soon reached at which formation of pus commences, and the child becomes permanently deaf from bursting of the membrane, or the still more serious complication occurs of extension of inflammation to the membranes of the brain, an occurrence for which every facility is arranged by the intimate communications which, in the infant especially, exist for such an issue, convulsions, coma, and death rapidly succeed."

Now, the point I wish to emphasize is this: the pain is not what we vaguely term neuralgia; it is a definite trophic change, an inflammation taking place in the deeper seated tissues of the ear; the gums are lanced and thereby reflex irritation is lessened.

But a child has by no means escaped ear trouble arising from the teeth if it has safely passed over the period of their evolution. External otitis has been distinctly traced in children to the presence of a carious tooth, and even in later life a decaying tooth will indicate its presence by prolonged earache and will even establish an otorrhœa. The first example I gave you was of this kind.

As another example of trophic or nutrition lesions of a more trivial kind I may mention the following, recorded by Mr. Hilton:

"A person was brought to me by a surgeon, suffering very great pain on the left side of his face. He was much exposed to the weather and suffered a great deal in consequence. He had taken a good many things to cure the 'neuralgia' as it was termed. I observed that he wore a wig, and I asked the reason. He said, curiously enough the hair on my left temple has all turned gray. I did not like to have black hair on one side and gray on the other, so I had my head shaved and wear a wig. Upon examining his mouth, I found he had a decayed molar tooth (rather painful) on the left side of the lower jaw, supplied by the third division of the fifth nerve. When this second lower molar was extracted the neuralgic pain very nearly ceased. I have not seen the patient since, so cannot say whether or not the hair has recovered its color. All I can say is, it was stated to me that during the time he was suffering extreme pain on the left side, the hair over the temporal region became nearly white, the difference in color suggesting to me some structural deterioration, and to the patient the propriety of having his head shaved and wearing a wig."

Another case is related where the constant presence of fur on one-half of the tongue caused a patient to seek advice. The patient was found to have a decayed molar tooth on the same side as furred tongue. The tooth was removed, and a fortnight afterwards all the fur had subsided.

Quite a different explanation has been put forward of this unilaterally furred condition of the tongue, viz., that the half of the jaw in which the diseased tooth is situated is but little used in mastication, and hence no removal of epithelium of the tongue takes place by the friction of the food on that side. This objection is completely overruled by the following:—It is shown that the tongue was furred only over the distribution of the lingual gustatory to the anterior part of the tongue, whereas it is clear that if the furring had resulted merely from a want of use of that part of the jaw, the fur would not have been limited to the anterior, but likewise have affected the posterior part of the tongue; this part, which receives its nerve supply from the glosso-pharyngeal nerve, may sometimes be seen to be furred in cases of inflamed tonsils, which also receive branches from the glosso-pharyngeal.

Mr. Hyde Salter relates the case of a healthy young woman, from Bournemouth, who consulted him on account of an ulcer, about the size of a shilling, on the side of the neck; this had commenced some twelve months before as a painful red spot and had been treated by applications of every kind without the slightest improvement in the condition of the ulcer. Mr. Salter examined the mouth, and found the wisdom (lower) on the same side of the jaw as the ulcer to be in an advanced stage of caries; this tooth was promptly extracted and within a fortnight after, the ulcer was completely cured and remained a firm cicatrix ever after.

In all the examples I have given you a definite nutritive change has been found to exist in the irritated area, whether resulting in such slight changes as the turning grey of hair, a condition found normally later in life in the production of an excessive amount of ill-formed epithelium, or a condition of hyperæmia, inflammation, and suppuration, as exemplified in the ear disease, up to the local destruction or death of tissue resulting in an ulcer.

The question now before us is to find the solution of the various symptoms detailed. Taking the case in which inflammation of the external ear resulted from the cutting of a tooth, we have the phenomena of pain, inflammation, and suppuration in an organ widely separated from the recognized exciting cause, and with no obvious intercommunication between the two except through the medium of nerve fibres; the simple continuity of sensori-motor nerves is insufficient to produce the conditions under review. The explanation will be found in the relation of the vaso-motor nerves and the functions which it is their office to fulfill. These vaso-motor nerves have the power of altering the size of the arteries to which they are distributed, and by this means can increase the supply of blood entering the part to which the irritation is applied, or when they act so as to contract the vessels greatly diminish the amount of blood circulating.

The action of the sympathetic nerve just described can be clearly shown in a transparent tissue, such as is found in the ear of a white rabbit. Section of the sympathetic supplying the ear causes a deep blush to suffuse the whole organ, showing an increase of blood supply from an enlargement of the calibre of the blood-vessels. On the other hand, irritation of the cut end of the sympathetic by an electric discharge immediately produces an unnatural pallor in the pinna of the ear. Now, a considerable portion of the blood supply of the membrane of the drum is derived from an artery that leaves the internal carotid and proceeds by a very short course to its destination, being thus closely connected with a large arterial trunk; this small artery possesses very favorable circumstances for a speedy augmentation of its blood supply. Now, the sympathetic upplying the carotid plexus comes largely from the otic ganglion, which ganglion controls the circulation of this part. On the other hand, the inferior dental nerve supplying the teeth and gums also communicates with this ganglion. We thus arrive at a direct channel of nerve communication through the otic ganglion, between the source of irritation, the tooth, and the vascular supply of the drumhead. Therefore, any irritation of the nerve in the vicinity of the tooth passes upwards to the otic ganglion, from which it is reflected to the nerves governing the blood supply of the drum, the vessels of which become largely distended, and if the irritation be sufficiently prolonged, an effusion takes place into the tissues of the drum, and finally, the formation of pus results. This disturbance of the vessels soon extends deeper and pus distends the internal ear, causing perhaps rupture of the drum, or terminating in convulsions and death. It will thus be clear that earache, under whatever circumstances it occurs, whether resulting from tooth diseases or any other cause, is never a trivial accident to be treated with a boiled onion or warm oil. The correct treatment may be roughly summarized under two heads: 1st. Removal of the cause, as by lancing a swollen gum or removing a decaying tooth. 2d. By the treatment of the effects, which will vary according to the stage at which they have arrived. Thus, if there is only a hyperæmia, apply a leech inside the pinna of the ear, and give small doses of tincture of aconite frequently, to diminish the heart's action, following this up with syringing the ear with warm water. If the disease has gone on to the formation of matter, we must follow the ordinary rule and give exit to it either by puncturing the drum or opening the mastoid cells.—*British Journal of Dental Science.*

